# A 75-ps Gated CMOS Image Sensor with Low Parasitic Light Sensitivity

**DOI:** 10.3390/s16070999

**Published:** 2016-06-29

**Authors:** Fan Zhang, Hanben Niu

**Affiliations:** Key Laboratory of Optoelectronic Devices and Systems of Education Ministry and Guangdong Province, College of Optoelectronic Engineering, Shenzhen University, Shenzhen 518060, China; zhangfan3@email.szu.edu.cn

**Keywords:** CMOS image sensor (CIS), gated imager, snapshot imager, ultra-fast global shutter, framing camera, low parasitic light sensitivity, high shutter efficiency

## Abstract

In this study, a 40 × 48 pixel global shutter complementary metal-oxide-semiconductor (CMOS) image sensor with an adjustable shutter time as low as 75 ps was implemented using a 0.5-μm mixed-signal CMOS process. The implementation consisted of a continuous contact ring around each p+/n-well photodiode in the pixel array in order to apply sufficient light shielding. The parasitic light sensitivity of the in-pixel storage node was measured to be 1/8.5 × 10^7^ when illuminated by a 405-nm diode laser and 1/1.4 × 10^4^ when illuminated by a 650-nm diode laser. The pixel pitch was 24 μm, the size of the square p+/n-well photodiode in each pixel was 7 μm per side, the measured random readout noise was 217 e^−^ rms, and the measured dynamic range of the pixel of the designed chip was 5500:1. The type of gated CMOS image sensor (CIS) that is proposed here can be used in ultra-fast framing cameras to observe non-repeatable fast-evolving phenomena.

## 1. Introduction

Fast gated or global shutter cameras with shutter time at a level of tens of picoseconds are widely used in the observation of fast-evolving phenomena, including repeatable and non-repeatable processes. Traditionally, micro-channel plate (MCP)-based gating cameras are used in range imaging systems (time-of-flight depth cameras) and wide-field fluorescence-lifetime imaging microscopy to observe repeatable fast evolving phenomena. In recent years, a large number of solid-state devices have been developed for such applications [1,2,3,4,5].

Currently, MCP-based gated cameras are almost the only type of receive-only 2D imaging device used in applications that require the observation of non-repeatable fast-evolving phenomena, with a time resolution as little as approximately 35 ps [6]. Such applications include plasma expansion dynamics research, charged particle accelerator diagnosis, optical time-of-flight measurements of fast moving objects, and high-resolution photo-acoustic imaging. However, some other successful efforts have also been presented for these purposes that use streak cameras [7] or that rely upon light-absorption-induced modulation of the optical refractive index of a semiconductor sensor medium [8].

A pulse-dilation enhanced gated optical imager can achieve a time resolution of approximately 5 ps [9,10,11], which is an overwhelmingly high speed for receive-only 2D imaging. The drawback of such a device is that it is bulky in size, sensitive to magnetic fields, and relatively low in spatial resolution.

Very fast global shutter complementary metal-oxide-semiconductor (CMOS) readout test chips with on-chip photodiodes have also been implemented [12]. Tests to measure the minimum exposure time when using on-chip photodiodes have achieved results of approximately 200 ps [13], but without any reports on the successful implementation of the sensor chip it requires, their use in practical applications is still limited. To the best of the authors’ knowledge, no reports have been given on the parasitic light sensitivity of the global shutter CMOS readout chip while using the on-chip photodiodes.

If the parasitic light sensitivity does not meet requirements, artifacts will be captured within an image from bright moving objects or light spots after exposure and before the readout [14].

In this paper, the authors present the detailed design, test methods, and results of a low parasitic light sensitivity 40 × 48 pixel gated CMOS image sensor that is sensitive to visible and near ultraviolet light with a shutter time as low as 75 ps, which is manufactured using a 0.5-μm 2-poly 3-metal polycide mixed-signal CMOS process. The type of CMOS image sensor proposed in this paper can be used in ultra-fast framing cameras to observe single-shot fast-evolving phenomena.

## 2. Pixel Circuit Design

**Figure 1:**
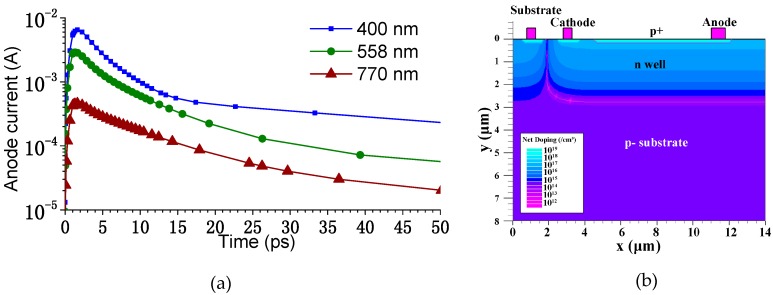
(**a**) The simulated transient response of the p+/n-well photodiode at three different wavelengths; (**b**) The structure of the photodiode used in the transient simulation.

In order to obtain the desirable fast photo response, p+/n-well photodiodes are used in the pixel array. Semiconductor processes and device simulations have shown that a small-sized p+/n-well photodiode manufactured using this process has an impulse response time that is shorter than approximately 5 ps, for visible light or near ultraviolet, at a bias of 5 V. Figure 1a shows the simulated photocurrent response of the small-sized p+/n-well photodiode at a 5-V bias after illumination by 1 ps short light pulses with 0.2 pJ of energy. The software used in the device simulation was Silvaco Atlas, and Figure 1b shows the structure and the doping profile of the photodiode used in the device simulation. A wavelength of 558 nm was used in the simulation, which is the maximum emission of a bright ultra-fast scintillator (n-C_6_H_13_NH_3_)_2_PbI_4_, which is in a natural multiple quantum well (MQW) structure and has a decay component of 390 ps (30%) at room temperature [15]. X-ray or electron sensitivity can be achieved by coupling the proposed image sensor to this type of bright ultra-fast scintillator screen by microscopy.

**Figure 2:**
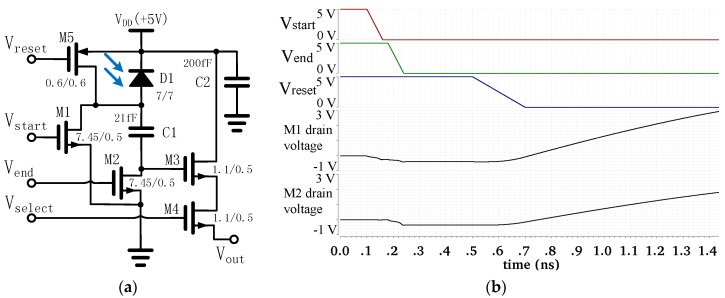
(**a**) The pixel circuit schematic; (**b**) The timing chart of the pixel and the simulated results of the drain voltage of M1 and M2.

The circuit diagram for a single pixel is shown in Figure 2a. In the design, V_reset_, V_start_, and V_end_ are set to the same value as V_DD_, and V_select_ is set to ground when waiting for the trigger signal. Thus, only the transistors M1 and M2 are turned on. Once triggered, V_start_ and subsequently V_end_ are pulled down to ground in the sequence, within a time interval that is slightly shorter than the exposure time. During exposure, when M1 is turned off and M2 is still on, part of the photo-induced charge is stored on the polysilicon-insulator-polysilicon (PIP) capacitor C1. After exposure, all five transistors of the pixel are turned off. After approximately 14 nanoseconds, V_reset_ is pulled down to ground, thus pulling up the anode of the photodiode to V_DD_ and forming the final signal voltage on the gate of M3 for the read-out.

A timing chart for V_start_, V_end_, V_reset_, and the simulated results of the drain voltages of M1 and M2 is shown in Figure 2b. In the simulation, a 6.25-fF capacitor representing the parasitic capacitance was added between the bottom plate of C1 and the ground. Figure 2b shows that the M2 drain voltage drops to around −0.396 V after V_end_ has been pulled down to ground. This value is acceptable as the simulated leakage current of the 0.396-V forward-biased p− substrate/n+ drain diode of M2 is only 216 pA at room temperature. The relatively large parasitic capacitance between the bottom plate of C1 and the grounded p− substrate is the key to keeping the M2 drain voltage from dropping deeper, while it also restricts the sensitivity of the proposed image sensor.

In more recently developed global shutter CMOS image sensors, a photodiode substrate and an in-pixel storage node substrate is interconnected by microbumps to achieve excellent parasitic light sensitivity [14]. In an ultra-fast gated CMOS image sensor, this type of strategy is not the best choice as the parasitic capacitance of the microbump interconnections is too large. Instead, sufficient light shield, and some shield for the carriers, is applied to a single-chip CMOS image sensor to achieve a low enough level of parasitic light sensitivity. In the proposed CMOS image sensor, the entire area in the pixel array, with the exception of the photodiodes, is shielded by the top metal layer in order to achieve high shutter efficiency. Furthermore, for each pixel, a continuous contact ring is included in the design, which is in contact with the n+ active area within the photodiode n-well and surrounding the photodiode p+ active area, in order to achieve superior light shielding efficiency. The anode of the photodiode (the p+ area) is led out by metalized polysilicon through a small opening on the contact ring. There are also continuous via rings between the metal layers (1,2) and (2,3) surrounding the photodiode without any openings. Although using continuous contact rings or via rings in the circuit violates the topological design rule from the foundry, the proposed design works well. It is based on the 0.5-μm CMOS process without any changes to the default process parameters. The 0.5-μm CMOS process that is used to fabricate the proposed chip does not include any chemical mechanical planarization (CMP) processing.

**Figure 3:**
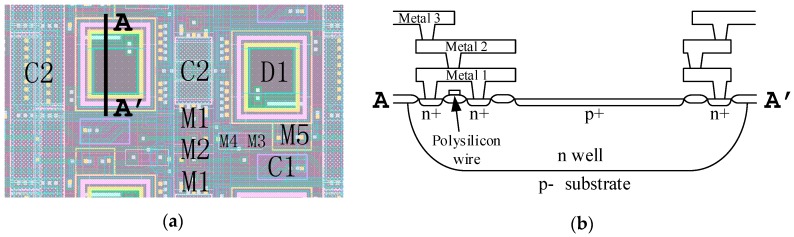
(**a**) The layout of two pixels; (**b**) Cross-sectional diagram of the p+/n-well photodiode in a pixel.

The layout of a couple of pixels in the pixel array is shown in Figure 3a, and a cross-sectional diagram of the photodiode in the pixel is shown in Figure 3b. Transistors M1 and M2 of the pixels in the even and odd columns share the same active areas. Thus, the drain of transistor M2 is far away from the nearest contact opening, which helps provide sufficient light shielding to the drain of transistor M2. This approach also simplifies the layout of the vertical clock tree in the pixel array.

In order to minimize the drain capacitance, both gates of transistors M1 and M2 are configured in a square annular structure. This also provides some shield for the photoelectrons for the drain of M2 and increases the shutter efficiency. The authors also used several depletion NMOS capacitors with an approximate total value of 200 fF in each pixel for power-decoupling purposes.

## 3. Limitations of Shortest Shutter Time

**Figure 4:**
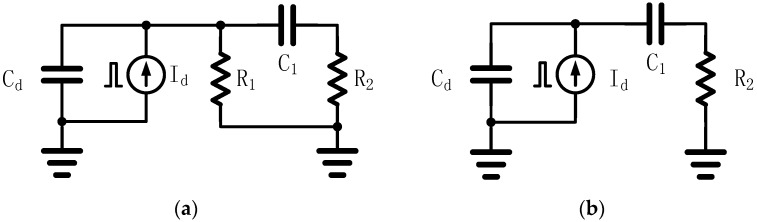
Simplified circuit model of a pixel. (**a**) Before exposure. (**b**) During exposure.

Figure 4a shows a simplified circuit model of a pixel before exposure at the moment when transistors M1 and M2 are both on. C_d_ includes the capacitance from the photodiode D1 and the transistors M1 and M5. C_1_ represents the capacitance of the sampling capacitor C_1_ in Figure 2a. R_1_ and R_2_ represent the on-state resistance of the transistors M1 and M2, respectively. It can be assumed that the pulse current source I_d_ emits a short enough current pulse with a total charge of *Q_p_* before exposure. The time interval between the current pulse and the start of exposure is *t*_1_, and *Q*_1_ is the charge on capacitor *C*_1_ after the shutter has remained in the “open” state, as shown in Figure 4b, for sufficient time. For simplicity, let *C* = *C_d_* = *C*_1_, and then *Q*_1_ can be expressed as

(1) $$Q_{1}=\frac{\left(2R_{1}-R_{2}\right)\sinh(\tau t_{1})+2R_{1}R_{2}C\tau \cdot \cosh(\tau t_{1})}{4R_{1}R_{2}C\tau }\cdot e^{-\frac{2R_{1}+R_{2}}{2R_{1}R_{2}C}t_{1}}\cdot Q_{p}$$

(2) $$\tau =\frac{\sqrt{4{R_{1}}^{2}+{R_{2}}^{2}}}{2R_{1}R_{2}C}$$

*Q*_1_ will decline as *t*_1_ increases. In the proposed design, *R*_1_ = *R*_2_ = 1413 Ω, and we assume *C* = 21 fF. Let *t*_half1_ be the value of *t*_1_ when *Q*_1_ has decreased to half of its maximum value. For the values given above, *t*_half1_ = 31.5 ps.

Figure 4b shows the simplified circuit model of a pixel during exposure, when transistor M1 is off and M2 is still on. It can be assumed that the current source I_d_ emits a short enough current pulse with a total charge *Q_p_* during the shutter “open” state. If *t*_2_ denotes the time interval after the current pulse and *Q*_2_ denotes the charge on capacitor C_1_ at time *t*_2_, *Q*_2_ can be expressed as

(3) $$Q_{2}=\frac{C_{1}}{C_{1}+C_{d}}\left[1-e^{-\frac{C_{1}+C_{d}}{R_{2}C_{1}C_{d}}t_{2}}\right]Q_{p}$$

*Q*_2_ will increase after the current pulse and eventually reaches a maximum value. Let *t*_half2_ be the time after the current pulse when *Q*_2_ reaches half of its maximum value; *t*_half2_ can then be expressed as

(4) $$t_{half2}=\ln(2)\frac{R_{2}C_{1}C_{d}}{C_{1}+C_{d}}$$

In the proposed design, *C*_1_ = 21 fF, *R*_2_ = 1413 Ω, and the simulated value of *C*_d_ is 27 fF with a 5-V power supply, giving *t*_half2_ = 11.7 ps. The shortest shutter time of the proposed design should therefore be longer than *t*_half1_ + *t*_half2_, i.e., 43.2 ps.

## 4. Sensor Chip Architecture

**Figure 5:**
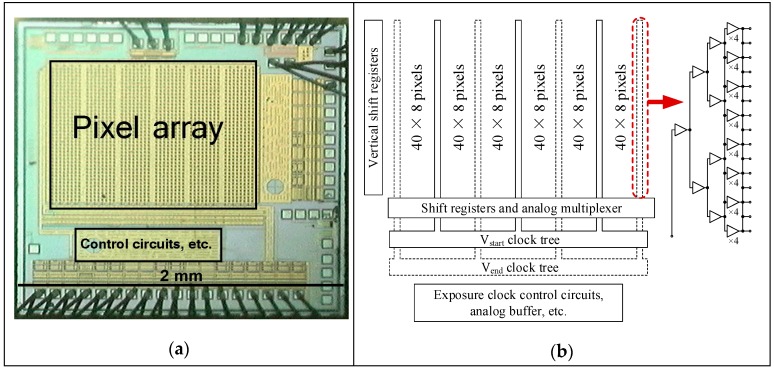
(**a**) Micrograph of the designed image sensor. (**b**) Structural diagram of the designed image sensor; simplified schematic of a single vertical component in the clock trees.

Figure 5a shows a micrograph for the designed image sensor, and Figure 5b illustrates the circuit architecture of the sensor. The exposure control signals V_start_ and V_end_ can be configured to be directly controlled by an external digital input, or alternatively the exposure process can be triggered using an external digital signal. When the exposure process is triggered by an external signal, the time between the falling edge of V_start_ and V_end_ signal (roughly the exposure time) is controlled by a voltage-controlled delayer, which is located in the exposure clock control circuits at the bottom of the chip.

The exposure control signals V_start_ and V_end_ are firstly distributed across the horizontal components [4,5], then across the vertical components of the clock trees, and finally to the pixels. The vertical components of the clock trees are placed in the pixel array by pruning one row of pixels after every eight rows, as shown in Figure 5b. The three even and the four odd vertical components of the clock trees belong to the V_start_ and the V_end_ signals, respectively. A simplified schematic of the single vertical components of the clock trees is shown in the right part of Figure 5b. These clock trees consist of fast falling-edge digital buffers [12] and distributed power decoupling capacitors. This type of design of clock trees can be easily extended to large-format gated CMOS image sensors.

Since balanced clock trees with fast falling-edge digital buffers are used in the exposure control signal distribution in both the horizontal and vertical directions, and the output of the final nodes of all vertical components of the V_start_ clock tree are connected together as shown in the right part of Figure 5b, as are the horizontal components and the V_end_ clock tree, the exposure signal skew should be relatively small compared with the shortest shutter time of the small designed image sensor.

The image signal from the pixels is first multiplexed by an analog multiplexer to a voltage shifter, and it is then buffered by an on-chip analog buffer and eventually drives an off-chip analog-to-digital (A/D) converter.

## 5. Test Methods and Results

A test board connected to a PCI digital data acquisition board was used to test the designed chip. A 12-bit A/D converter chip operating at a 5-V input range was used on the test board. The highest speed achievable by the digital data acquisition board when operating bi-directionally is 10 M samples per second. This speed limits the A/D converter clock frequency and the sampling rate to a maximum of 5 M samples per second.

**Figure 6:**
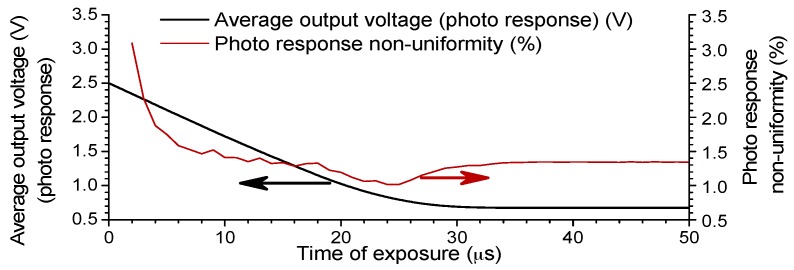
The measured photo response curve and the photo response non-uniformity of the designed image sensor.

Figure 6 shows the measured photo response curve and the photo response non-uniformity (PRNU) between pixels of the proposed image sensor. The photo response curve and the PRNU were obtained by varying the exposure time, while keeping a constant uniform illumination by a blue LED. The measured PRNU for the selected area at half of the saturated voltage for all columns, odd columns only and even columns only was 1.39%, 1.42%, and 1.28%, respectively. This is normal and the difference in the pixel layouts for the odd and even columns show no significant influence on the PRNU.

**Figure 7:**
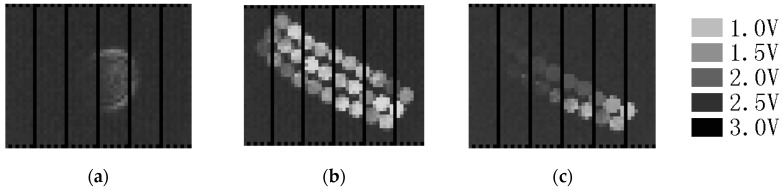
Images captured by the designed image sensor. (**a**) Image of the defocused laser spot used in the small signal responsivity measurement; (**b**) Image of the fiber bundle at a 17 ns shutter time. (**c**) Image of the fiber bundle at a 30 ps simulated shutter control signal delay.

To measure small signal responsivity or the charge-to-voltage gain of the designed image sensor, the central area of the pixel array was illuminated by a defocused 405-nm wavelength continuous-wave (CW) diode laser spot, as shown in Figure 7a. The difference between the measured total supply current of the chip when the 405-nm laser was on and off was the measured total photocurrent. During such measurements, the pixel array was in a state of waiting for the trigger signal, and V_select_ of all pixels was set to ground, so that the output analog buffer remained in the same state. The measured small signal responsivity of the designed chip was 1.47 μV/e^−^. The linear range of the output signal was from 2.5 V to about 0.7 V, so the full capacity of the pixel was around 1,200,000 e^−^. The measured random readout noise of the output signal was 475 μV rms. The quantization noise of a 12-bit readout with a 5-V full range is 352 μV rms. Thus, the random readout noise of the designed chip was 319 μV rms, which is equivalent to 217 photoelectrons generated by the photodiode. Therefore, the dynamic range of the designed chip was about 5500:1.

The measured parasitic light sensitivity of the in-pixel storage node was very low when illuminated by a continuous-wave diode laser with a peak wavelength of 405 nm. The parasitic light sensitivity was measured by comparing the following two images. For the first image, the shutter time was set to approximately 300 ps. The exposure to the 405-nm diode laser lasted 0.5 s after the shutter was closed, and the captured image was then read out. The second image was taken by setting the shutter time to 100 ns and read out immediately after the shutter was closed. The dark image taken with the laser off was subtracted from both images to eliminate the output signal bias and fixed pattern noise. The resulting two images were then used to calculate the parasitic light sensitivity. The final measured parasitic light sensitivity when illuminated by a 405-nm diode laser was 1/8.5 × 10^7^.

The parasitic light sensitivity when illuminated by a 650-nm continuous-wave diode laser was measured using a similar method, and the measured value was 1/1.4 × 10^4^.

The measured leakage signal in a dark environment after the global shutter was closed was 0.7 V/s. According to the simulation, this leakage signal value is equivalent to a leakage current of approximately 22 fA on the storage node in the pixel.

**Figure 8:**
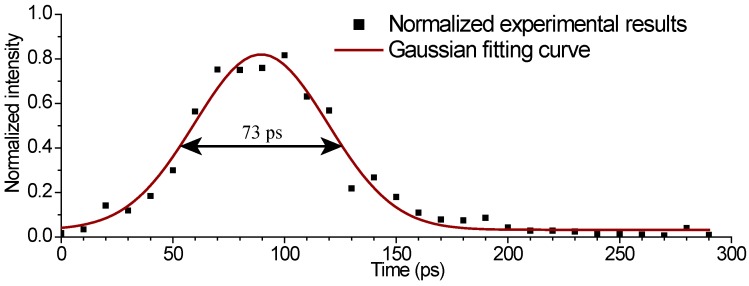
Exposure curve of the designed image sensor.

The shortest shutter time (fastest shutter speed, best temporal resolution) of the designed chip was measured using a frequency-doubled 400-nm wavelength Ti:sapphire laser system with a 130-fs pulse width. The 400-nm laser flash was used to uniformly illuminate a fiber cable. The cable was composed of 30 silica fibers of different lengths [11]. The difference in length between adjacent fibers in the fiber cable was 2.0 mm. The output port of the fibers was imaged on the image sensor using the lens. During the shutter time measurement, the image sensor was triggered by a biased p-i-n photodiode outside the chip. Figure 7b shows the image that was obtained at a 1-V exposure time control voltage, which corresponds to a shutter time of 17 ns, whereas Figure 7c was obtained at a 4-V exposal time control voltage, which corresponds to a 30 ps simulated shutter control signal delay. The two images were used to obtain a normalized exposure curve, as shown in Figure 8. The measured shortest shutter time of this camera was less than 75 ps.

The characteristics and measurement results of the designed image sensor and a comparison with prior works are summarized in Table 1.

## 6. Discussion

A measured leakage signal of 0.7 V/s in a dark environment is too large compared to the readout time of a large-format imager when there are 5-M samples being read out per second. Therefore, either the readout speed needs to be increased, or the leakage current needs to be lowered for an imager with much more pixels. Methods such as cooling or improving the pixel circuit design can be used to lower the leakage current.

The measured minimum shutter time of 75 ps is much larger than the calculated value of 43.2 ps, and the exposure curve shown in Figure 8 seems to be symmetric. This is as expected, since the shutter time is limited mainly by the fall time of the exposure control signals V_start_ and V_end_ driving the gates of M1 and M2, and not by the intrinsic minimum shutter time of the pixel circuit.

The parasitic light sensitivity that is measured when a 650-nm diode laser is used for illumination is much higher than that obtained using a 405-nm diode laser. This is due to the fact that the absorption depths of light at 405 nm and 650 nm in intrinsic silicon is approximately 0.12 μm and 3.56 μm, respectively [16]. Therefore, much more photoelectrons are generated in the p− substrate under the photodiode when it is illuminated by the 650-nm light, and some of these photoelectrons drift to the n+ drain of the transistor M2, although the p-well of transistor M2 provides some shield to the photoelectrons generated in the p− substrate [17]. Therefore, placing the p-well of transistor M2 in a deep n-well isolated area may provide considerable improvement to the shutter efficiency.

Since the exposure signal skew is relatively small compared with the shortest shutter time in the small designed image sensor, and there is a lack of pixels with skew test circuits [12] in the pixel array, it is hard to measure the exact exposure signal skew. Precise measurement may be possible in the future using an ultra-fast gated CMOS image sensor based on a similar design, but with a much larger imaging area.

## 7. Conclusions

For this paper, a 40 × 48-pixel ultra-fast global shutter CMOS image sensor was designed and manufactured using a 0.5-μm mixed-signal CMOS process. The measured parasitic light sensitivity for a 405-nm diode laser was 1/8.5 × 10^7^, which is comparable to MCP-based gated cameras and is low enough for most applications. The measured shutter time can be as short as 75 ps, and the measured dynamic range of the pixel of the designed chip was 5500:1, which is no worse than MCP-based picosecond framing cameras that are currently used [18]. The authors are confident that further significant improvements can be made to the proposed design’s temporal resolution through the combined use of more advanced CMOS processes such as advanced silicon-on-insulator (SOI) CMOS technologies and by overdriving the gates of M1 and M2 immediately before and during the exposure process.

## Figures and Tables

**Table 1:** Comparison with previous ultra-fast gated CMOS image sensors.

Reference	JSSC 2008 [12] SPIE 2012 [13]	SPIE 2012 [13]	JSSC 2016 [3]	This Work
Design aim	Test readout chip for ultra-fast gated X-ray imager	Readout chip for ultra-fast gated X-ray imager	Fluorescence lifetime imaging	Test ultra-fast gated imager for visible light
Supply voltage	1.8 V	1.8 V	--	5 V
Process	0.18-μm CMOS	0.18-μm CMOS	0.11-μm CIS CMOS	0.5-μm CMOS
Chip size	3 mm × 3 mm	> 15 × 15 mm	7.0 mm × 9.3 mm	2 mm × 2 mm
Resolution	64 × 64 pixels	512 × 512 pixels	256 × 512 pixels	40 × 48 pixels
Pixel pitch	30 μm	30 μm	11.2 μm × 5.6 μm	24 μm
Photodiode aperture area	--	--	~ 10.5 μm^2^	6.9 μm × 6.9 μm
Power consumption	125 mW	--	540 mW	50 mW
Fixed pattern noise (rms)	9 mV	--	0.12 e^−^ (vertical)	23.3 mV
Random readout noise	115 e^−^	--	1.75 e^−^	475 μV
Random readout noise (rms, with quantization noise subtracted)	--	--	--	217 e^−^ (319 μV)
PRNU	--	--	--	1.4%
Full capacity	310, 000 e^−^	--	2,700 e^−^	1,200,000 e^−^
Small signal responsivity	11 μV/e^−^	--	85 μV/e^−^	1.47 μV/e^−^
Output swing	0.8 V	--	0.3 V	1.8 V
Leakage signal (global shutter closed)	< 125 fA	--	--	22 fA (0.7 V/s)
Parasitic light sensitivity	--	--	1/16.7 (472 nm)	1/8.5 × 10^7^(405 nm) 1/1.4 × 10^4^(650 nm)
Shortest shutter time	200 ps	250 ps	180 ps (374 nm)	75 ps
